# m6A RNA modification pathway: orchestrating fibrotic mechanisms across multiple organs

**DOI:** 10.1093/bfgp/elae051

**Published:** 2025-01-05

**Authors:** Xiangfei Huang, Zilu Yu, Juan Tian, Tao Chen, Aiping Wei, Chao Mei, Shibiao Chen, Yong Li

**Affiliations:** Department of Anesthesiology, The First Affiliated Hospital, Jiangxi Medical College, Nanchang University, 17 Yongwaizheng Street, Donghu District, Nanchang 330006, China; Queen Mary School, Medical College, Nanchang University, 1299 Xuefu Road, Honggutan District, Nanchang 330031, China; Department of Anesthesiology, The First Affiliated Hospital, Jiangxi Medical College, Nanchang University, 17 Yongwaizheng Street, Donghu District, Nanchang 330006, China; Department of Anesthesiology, The First Affiliated Hospital, Jiangxi Medical College, Nanchang University, 17 Yongwaizheng Street, Donghu District, Nanchang 330006, China; Department of Anesthesiology, The First Affiliated Hospital, Jiangxi Medical College, Nanchang University, 17 Yongwaizheng Street, Donghu District, Nanchang 330006, China; Department of Anesthesiology, The First Affiliated Hospital, Jiangxi Medical College, Nanchang University, 17 Yongwaizheng Street, Donghu District, Nanchang 330006, China; Department of Anesthesiology, The First Affiliated Hospital, Jiangxi Medical College, Nanchang University, 17 Yongwaizheng Street, Donghu District, Nanchang 330006, China; Department of Anesthesiology, The First Affiliated Hospital, Jiangxi Medical College, Nanchang University, 17 Yongwaizheng Street, Donghu District, Nanchang 330006, China

**Keywords:** m6A modification, organ fibrosis, RNA epitranscriptomics, fibrotic diseases, therapeutic targets

## Abstract

Organ fibrosis, a common consequence of chronic tissue injury, presents a significant health challenge. Recent research has revealed the regulatory role of N6-methyladenosine (m6A) RNA modification in fibrosis of various organs, including the lung, liver, kidney, and heart. In this comprehensive review, we summarize the latest findings on the mechanisms and functions of m6A modification in organ fibrosis. By highlighting the potential of m6A modification as a therapeutic target, our goal is to encourage further research in this emerging field and support advancements in the clinical treatment of organ fibrosis.

## Introduction

In response to organ injuries, a cascade of sequential yet interrelated stages occurs, encompassing inflammation, proliferation, and remodeling [1]. Unfortunately, even in tissues with significant regenerative potential, such as the liver, wound repair cannot solely rely on the regeneration of parenchymal cells while disregarding the role of connective tissues [2]. Under ideal conditions, the excessive accumulation of extracellular matrix (ECM) resulting from acute organ injury is broken down and eliminated with the assistance of matrix metalloproteinases, leading to the restoration of both structure and function [3]. Nevertheless, repeated insults can disrupt the finely balanced ECM environment, disturbing the normal wound-healing process and resulting in progressive scarring [4]. Significantly, the formation of pathological scars during the tissue repair process, known as fibrosis, is characterized by the excessive synthesis, production, and deposition of ECM proteins such as collagen and fibronectin. This process plays a crucial role in organ failure and disease progression [5, 6]. Fibrosis has been implicated in the pathogenesis of numerous organ pathologies, including those affecting the lungs, liver, heart, and kidneys [7]. It contributes to 45% of all deaths in developed countries [2].

Fibrosis is a condition that encompasses a multitude of cell populations, including those responsible for the pathological deposition of ECM and non-fibroblast cell types that regulate fibrosis. In the context of pulmonary fibrosis, mesenchymal cells are traditionally considered the primary source of ECM production. However, Zepp *et al.* [8] have reported that mesenchymal cells in distinct spatial locations exhibit unique regulatory functions. Similarly, hepatic stellate cells (HSCs) have heretofore been regarded as the principal collagen-producing cells in the liver [9]. However, recent studies by Dobie *et al.* [10] have provided a more detailed understanding of HSCs’ functions, indicating that only central vein-associated HSCs are the primary pathogenic collagen-producing cells. Concurrently, Ramachandran *et al.* [11] have identified a distinct subpopulation of macrophages characterized by TREM2^+^CD9^+^, which plays a pro-fibrogenic role in liver cirrhosis.

Moreover, other non-fibroblast cell types, including epithelial [12] and endothelial [13, 14] cells, have also demonstrated the ability to undergo phenotypic transitions. This process entails the loss of their distinctive morphology and markers, accompanied by the acquisition of myofibroblast-like characteristics. This phenomenon is recognized as epithelial-mesenchymal transition (EMT) and endothelial-to-mesenchymal transition. These transitions have been identified as contributory factors to the development of organ fibrosis. It is important to note that the various cell populations implicated in fibrosis do not operate in isolation; rather, their intricate intercellular interactions play a pivotal role in the pathogenesis of fibrosis [15].

In addition to the diverse cell populations, numerous signaling pathways are crucial in the fibrotic process. Interestingly, despite differences in the triggers that initiate disruption of the ECM in different organs, the process of fibrosis and the associated signaling pathways exhibit a high degree of conservation [5]. Among these pathways, the transforming growth factor-β (TGF-β) signaling pathway stands out as a critical contributor to the pathogenesis of fibrogenic responses in various organs [16]. However, it does not operate in isolation. Instead, it engages in extensive crosstalk with a network of other signaling pathways, including mitogen-activated protein kinase, Wnt/β-catenin, epidermal growth factor receptor, and more. Together, these pathways collectively govern the progression of fibrosis [17].

In the context of disease progression, the activation of specific signaling pathways and expression of cellular functions are intricately intertwined with the precise regulation of particular genes. Gene expression is regulated across regulation across multiple dimensions, encompassing epigenetic, transcriptional, post-transcriptional, translational, and post-translational modifications. The realm of RNA modifications, known as “epitranscriptomics,” plays a pivotal role in this intricate regulatory network. While DNA is known for its stability, RNA exhibits a wide range of structural diversity. This includes messenger RNA (mRNA) responsible for protein coding, as well as non-coding RNAs such as microRNA (miRNA), transfer RNA, short interfering RNAs, ribosomal RNA, and long non-coding RNAs (lncRNAs). Various RNA species have been found to contain modifications, with over 170 distinct RNA modification methods documented to date [18]. Of these modifications, N6-methyladenosine (m6A) is the most prevalent. m6A modifications can influence the structure, stability, degradation, and cellular interactions of target RNA molecules [19]. Therefore, m6A modifications play a crucial role in regulating various diseases, including cancer, neurological disorders, and metabolic conditions [20–22].

The development of RNA sequencing (RNA-Seq) has provided a powerful and high-throughput method for identifying methylated sites, offering unprecedented insights into the mechanisms underlying diseases associated with m6A modifications. In recent years, research has emerged uncovering the involvement of m6A modifications in organ fibrosis. This review aims to summarize and present the latest research findings on m6A modifications in organ fibrosis, with a focus on pulmonary, hepatic, renal, and cardiac fibrosis (Table 1). The purpose of this synthesis is to encourage further investigation into the mechanisms and treatments for organ fibrosis.

**Table 1:** The involvement of m6A regulators in organ fibrosis.

Organ	Type	m6A regulator	Ref.	
Lung	Writer	METTL3; METTL14; WTAP; ZC3H13; RBM15/15B; HAKAI	[23–31]	Deng, *et al.*, 2022; Zhou, *et al.*, 2022; Zhang, *et al.*, 2022; Huang, *et al.*, 2022; Zhang, *et al.*, 2022; Wang, *et al.*, 2022; Ning, *et al.*, 2022; Han, *et al.*, 2020; Ji, *et al.*, 2023
	Reader	YTHDC1–2; YTHDF1–3; IGF2BP1–3; FMR1; SND1	[23–27, 29, 32, 33]	Deng, *et al.*, 2022; Zhou, *et al.*, 2022; Zhang, *et al.*, 2022; Huang, *et al.*, 2022; Zhang, *et al.*, 2022; Ji, *et al.*, 2023; Zhang, *et al.*, 2021; Zhang, *et al.*, 2024
	Eraser	ALKBH5; FTO	[23, 26, 27, 34]	Deng, *et al.*, 2022; Huang, *et al.*, 2022; Zhang, *et al.*, 2022; Sun, *et al.*, 2022
Liver	Writer	METTL3; METTL14; METTL16; WTAP; ZC3H13	[35–44]	Gao, *et al.*, 2022; Feng, *et al.*, 2021; Li, *et al.*, 2022; Qu, *et al.*, 2021; Shu, *et al.*, 2022; Fan, *et al.*, 2022; Shu, *et al.*, 2021; Zhu, *et al.*, 2020; Feng, *et al.*, 2022; Chen, *et al.*, 2023
	Reader	YTHDF1; YTHDF3; YTHDC1	[41, 45–47]	Chen, *et al.*, 2023; Sun, *et al.*, 2022; Shen, *et al.*, 2022; Feng *et al.*, 2023
	Eraser	ALKBH5; FTO	[41, 47–50]	Chen, *et al.*, 2023; Shen, *et al.*, 2022; Yang, *et al.*, 2022; Chen, *et al.*, 2023
Kidney	Writer	METTL3	[51–55]	Jung, *et al.*, 2024; Ni, *et al.*, 2023; Tang, *et al.*, 2022; Liu, *et al.*, 2020; Liu, *et al.*, 2021;
	Reader	YTHDF1; IGF2BP2	[53, 56, 57]	Tang, *et al.*, 2022; Xing, *et al.*, 2022; Chen, *et al.*, 2023
	Eraser	ALKBH5; FTO	[57–61]	Chen, *et al.*, 2023; Ning, *et al.*, 2020; Li, *et al.*, 2022; Yang, *et al.*, 2022; Zang, *et al.*, 2022
Heart	Writer	METTL3	[62–66]	Ding, *et al.*, 2023; Zhou, *et al.*, 2022; Li, *et al.*, 2021; Zhuang, *et al.*, 2023; Cheng, *et al.*, 2023
	Reader	IGFBP2	[67]	Peng, *et al.*, 2022
	Eraser	FTO; ALKBH5	[68–71]	Li, *et al.*, 2022; Liu, *et al.*, 2022; Mathiyalagan, *et al.*, 2019; Zhuang, *et al.*, 2024.

Abbreviations: METTL: Methyltransferase-like protein; WTAP: Wilms tumor 1-associated protein; ZC3H13: Zinc finger CCCH domain-containing protein 13; RBM: The RNA-binding motif protein; YTHDC: YTH domain-containing protein; YTHDF: YTH domain-containing family protein; IGF2BP: Insulin-like growth factor binding protein; FMR1: Fragile X messenger ribonucleoprotein 1; SND1: Staphylococcal nuclease and tudor domain containing 1; ALKBH5: Alpha-ketoglutarate-dependent dioxygenase AlkB homolog 5; FTO: FTO alpha-ketoglutarate dependent dioxygenase.

## Brief view of m6A modification

**Figure 1:**
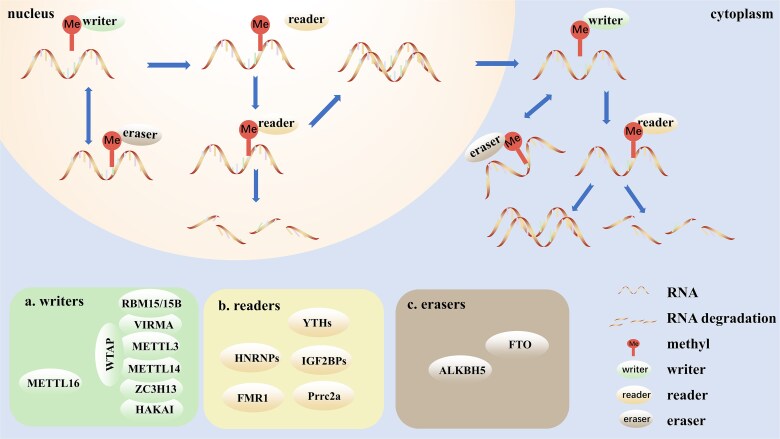
The mechanism and key regulators of m6A modification; (a). writers: methyltransferase complex composed of METTL3, METTL14, WTAP, VIRMA, ZC3H13, HAKAI, and RBM15/15B; METTL16. (b). readers: YTH, HNRNPs, IGF2BPs, FMR1, and Prrc2a. (c). erasers: FTO, ALKBH5.

The identification of m6A represents a significant milestone in RNA modifications. Its initial report dates back to 1974 by Desrosiers [72]. This modification is the most abundant among mRNA modifications and has been identified in various species, including mammals, yeast, plants, flies, and bacteria [19]. Importantly, m6A decoration is a reversible process regulated by three distinct categories of proteins known as “writers,” “erasers,” and “readers” (Fig. 1).

Within m6A modification, “writers” and “erasers” refer to methyltransferases and demethylases, respectively, which add and remove methylation from mRNA. Meanwhile, “readers” recognize m6A modifications [73]. The primary writer complex comprises multiple subunits, with methyltransferase-like 3 (METTL3) and methyltransferase-like 14 (METTL14) serving as the core components [74]. METTL3 is the catalytic subunit responsible for methyl group transfer, while METTL14 acts as a necessary RNA-binding platform [75]. Other subunits, such as Wilms tumor 1-associating protein (WTAP), Vir-like m6A methyltransferase-associated (VIRMA), zinc finger CCCH-type containing 13 (ZC3H13), HAKAI, and RNA-binding motif protein 15/15B (RBM15/15B), are essential for maintaining the stability, functionality, localization, and specificity of the writer complex [76–80]. In addition to the canonical m6A methyltransferase complex, METTL16 has gained attention as an m6A methyltransferase that prefers cytosolic localization, facilitating the translation of mRNA transcripts [81].

The group of “readers” encompasses widely recognized YT521-B homology (YTH) family proteins, heterogeneous nuclear ribonucleoproteins (HNRNPs), insulin-like growth factor 2 mRNA-binding proteins (IGF2BPs), fragile X mental retardation 1 (FMR1), and the recently disclosed proline-rich coiled-coil 2A (Prrc2a). These proteins affect the fate of RNA molecules, either enhancing the stability or facilitating the degradation of target transcripts [82–86]. Conversely, “erasers,” represented by m6A demethylases like fat mass and obesity-associated protein (FTO) and AlkB homolog 5 (ALKBH5), excise the methyl group from target RNA, conferring the m6A modification its reversibility and precise regulation [87, 88].

## m6A in organ fibrosis

### Pulmonary fibrosis

The utilization of gene sequencing technology has yielded unforeseen revelations in the exploration of disease mechanisms. Bioinformatics, which utilizes mathematical and statistical methodologies, is extensively used to predict disease occurrence and prognosis based on gene expression data. Several bioinformatics investigations employing gene expression data related to m6A regulatory proteins and methylated RNA immunoprecipitation sequencing (MeRIP-Seq) have delved into the intricate interplay between m6A modification and pulmonary fibrosis.

Regarding m6A writers, the methyltransferases METTL3, METTL14, and ZC3H13 have been identified as potential biomarkers for early diagnosis of pulmonary fibrosis and as prognostic indicators for patients with pulmonary fibrosis [23–25]. Furthermore, m6A readers, such as YTH domain-containing protein one and IGF2BP2–3, have also been identified as biomarkers for pulmonary fibrosis [24]. Additionally， the m6A eraser FTO has been associated with differences in immune cell infiltration and is crucial for the onset, progression, and prognosis of idiopathic pulmonary fibrosis (IPF) [26].

In addition to the m6A regulatory proteins, there has been significant focus on analyzing the downstream target genes influenced by m6A modification. Zhang *et al.* [27] investigated silica-induced pulmonary fibrosis and investigated the up-regulation of METTL3 and the downregulation of ALKBH5, FTO, YTHDF1, and YTHDF3. They utilized MeRIP-Seq and RNA-Seq to screen 18 genes with significant mRNA and m6A level changes. Functional and KEGG pathway analyses revealed that these screened genes predominantly enriched in immune response, phagocytosis, antigen processing and presentation, phagosome, lysosome, apoptosis, and other pathways. Similarly, Wang *et al.* [89] identified four m6A-related genes—*RBM11, RBM47, RIC3, TRAF5,* and *ZNF14*—using a multivariate Cox model, demonstrating their strong and stable predictive efficiency for IPF.

**Figure 2:**
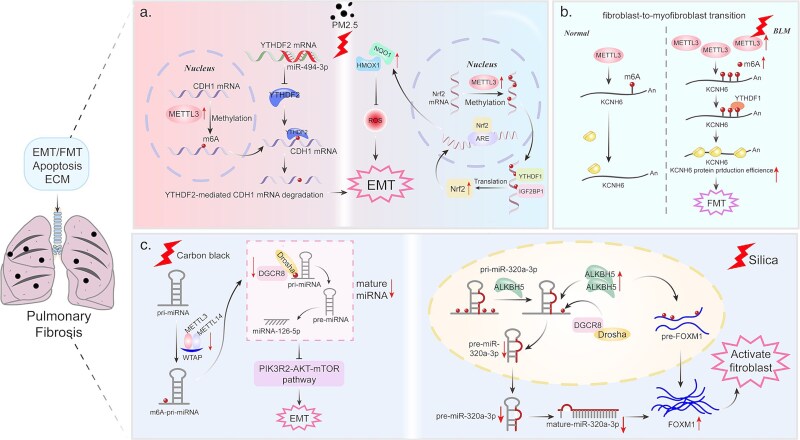
Mechanisms of m6A RNA Modification Involved in Pulmonary Fibrosis. (a) Stimulation by PM2.5 increases the METTL3-mediated CDH1 m6A modification recognized by YTHDF2 and inhibits E-cadherin expression and promotes the development of EMT. Increased METTL3-mediated m6A modification also can enhance the translation of the transcription factor Nrf, which attenuates PM2.5-induced pulmonary fibrosis. (b) In bleomycin (BML)-induced lung fibrosis, KCNH6 expression was enhanced by METTL3-mediated m6A modification in a YTHDF1-dependent manner. (c) Stimulated by inhalation of black carbon, the METTL3-mediated m6A modification of pri-miRNA-126 decreases and binding of DGCR8 is reduced, hindering miRNA-126 maturation, leading to activation of the PI3K-Akt–mTOR/PFKFB3 pathway and promotion of lung fibrosis. Induced by inhaled silica, the m6A eraser ALKBH5 demethylates pri-miR-320a-3p and prevents the binding of DGCR8, inhibiting miR-320a-3p maturation and leading to fibroblast activation.

Furthermore, several downstream target RNAs of m6A have been identified and associated with different processes in the context of pulmonary fibrosis. One of these targets is *CDH1*, which encodes the epithelial marker E-cadherin and undergoes a reduction during the EMT process. EMT is a crucial source of fibroblasts and contributes to tissue fibrosis [90]. Ning *et al.* [28] demonstrated that in a model of ambient airborne fine particulate matter-induced pulmonary fibrosis model that PM2.5 increases METTL3-mediated m6A modification of *CDH1*. This modified *CDH1* is then recognized by YTHDF2, which is negatively regulated by *miR-494-3p*. This recognition ultimately leads to the inhibition of E-cadherin expression, thereby promoting the progression of EMT. Whereas METTL3 has been reported to enhance the translation of the transcription factor Nrf, which can alleviate PM2.5-induced pulmonary fibrosis. *Nrf* is recognized by YTHDF1 and IGF2BP1 [29] (Fig. 2(a)).

In a process resembling EMT, myofibroblasts, originating from fibroblasts, represent a predominant source of collagen production. This transition is termed fibroblast-to-myofibroblast transition (FMT). Potassium Voltage-Gated Channel Subfamily H Member 6 (KCNH6), previously known for its role in glucose homeostasis and insulin secretion, has been found to regulate FMT. The expression of *KCNH6* is enhanced by METTL3-mediated m6A modification in a YTHDF1-dependent manner [32] (Fig. 2(b)).

In addition to mRNA, m6A modifications in non-coding RNAs (ncRNAs) assume a substantial role in the regulation of pulmonary fibrosis. The PI3K-Akt–mTOR/PFKFB3 pathway regulates pulmonary fibrosis by modulating fibroblast aerobic glycolysis and collagen synthesis [91]. In the context of pulmonary fibrosis, m6A modification of pri-*miRNA-126* decreases. This, coupled with a reduction in its binding with DGCR8, a key component involved in mRNA maturation [92], hinders the maturation of *miRNA-126*. Consequently, this leads to the activation of the PI3K-Akt–mTOR/PFKFB3 pathway [30] (Fig. 2(c)).

Similarly, Sun *et al.* [34] demonstrated that *miR-320a-3p* regulates fibrosis by targeting the 3′-untranslated region (UTR) of *FOXM1* mRNA. The m6A eraser ALKBH5 regulates the maturation of *miR-320a-3p* (Fig. 2(c)). Specifically, ALKBH5 demethylates *pri-miR-320a-3p* and prevents its binding with DGCR8, thereby inhibiting the maturation of *miR-320a-3p* (Fig. 2(c)). Additionally, Wang *et al.* [31] identified two circular RNAs, namely *hsa_circ_0000672* and *hsa_circ_0005654*, as participants in pulmonary fibrosis. This revelation was facilitated through the utilization of m6A-circRNA epitranscriptomic chips. The study suggested that METTL3 promotes SiO(2)-induced pulmonary fibrosis through m6A modifications in these two circRNA.

In addition to their canonical role in m6A regulation, RNA-binding proteins involved in m6A modification pathways have also been demonstrated to contribute pulmonary fibrosis independently of m6A modification. For example, YTHDC1 has been reported to mitigate cellular senescence-induced pulmonary fibrosis by facilitating the interaction between DNA topoisomerase 2-binding protein 1 (TopBP1) and Meiotic Recombination 11 (MRE11), thereby enhancing DNA damage repair [33]. These findings further expand the functional scope of m6A regulators in organ fibrosis.

### Hepatic fibrosis

Given the high prevalence of liver cirrhosis, extensive research has focused on m6A modifications in hepatic fibrosis. Using m6A-seq and RNA-seq methodologies, numerous studies have conducted screenings to identify distinct m6A methylation patterns associated with hepatic fibrosis. These patterns have demonstrated enrichment in various processes intricately linked to hepatic fibrosis [93, 94].

m6A modification assumes a dual role in the advancement and regression of hepatic fibrosis. Hepatic fibrosis progression is associated with oxidative stress and cytochrome metabolism, while fibrosis regression is intricately linked to immune response and apoptosis [95]. Chronic hepatitis B is among the most common causes of hepatic fibrosis. Gao *et al.* [35] demonstrated that METTL16 regulates the expression of the chronic hepatitis B-related gene *HLA-DPB1* and contributes to the progression of hepatic fibrosis. Significantly, TGF-β1, a pivotal constituent of the canonical fibrotic signaling pathway, emanates from activated Kupffer cells (KC) and propagates hepatic fibrosis. Activation of METTL3 and METTL14 amplifies *TGF-β1* mRNA levels in an m6A-dependent manner, further emphasizing the involvement of m6A modifications in hepatic fibrosis [36].

Since HSCs constitute the principal source of ECM and assume a pivotal role in hepatic fibrosis, considerable attention has been directed toward comprehending the regulatory role of m6A modification in HSC activation, thereby elucidating the mechanism underlying hepatic fibrosis.

Fan *et al.* [37] conducted a comprehensive analysis by examining 90 genes that displayed noteworthy alterations in both m6A and mRNA expression levels by applying m6A-seq and RNA-seq techniques. Their investigation substantiated distinctive expression patterns of m6A regulators, wherein the decreased expression of WTAP was determined to foster HSC activation, consequently instigating hepatic fibrosis. Conversely, the m6A methyltransferase METTL3 deficiency was shown to inhibit HSC activation in an m6A-dependent manner by targeting *Lats2*, a key modulator of the Hippo/YAP signaling pathway [38].

HSCs can also be activated by several types of immune cells, among which hepatic macrophages and their cross-talk with HSCs play a pivotal role in hepatic fibrosis. For example, exosomes derived from lipopolysaccharide (LPS)-induced macrophages have been observed to transfer miRNAs to promote HSC proliferation and activation during the progression of liver fibrosis [96]. It is noteworthy that METTL3 enhances the M1 polarization and inhibits M2 polarization of macrophages by directly methylating *signal transducer and activator of transcription 1* (*STAT1*) mRNA at its coding sequence and 3′-untranslated regions, which encodes the master transcription factor facilitating M1 macrophage polarization [97]. In a study conducted by Qu *et al.* [39], the online prediction tool Starbase was used to report the potential binding of METTL3 and the lncRNA *NEAT1*, which has been demonstrated to accelerate the progression of liver fibrosis [98]. Additionally, METTL3 has been shown to promote *NEAT1* expression in Kupffer cells and LPS-treated Kupffer cell exosomes, which can further enhance the activation of HSCs [40].

Several studies have also investigated the impact of m6A readers on HSC activation. For instance, Feng *et al.* [45] underscored the pivotal role of m6A modification in HSC activation and the excessive ECM production, which is mediated by the m6A reader YTHDF1. Furthermore, Chen *et al.* [41] reported the involvement of the circadian rhythm in the progression of liver fibrosis. They demonstrated that the downregulation of the liver clock gene *NR1D1* promoted persistent activation of HSCs and liver fibrosis induced by CCl4. Importantly, they found that the expression of *NR1D1* is regulated by m6A modification in a YTHDC1-dependent manner.

Furthermore, the m6A demethylase ALKBH5 has been implicated in the activation of HSCs through various downstream factors. Yang *et al.* [48] demonstrated, in a mouse model of CCl4-induced liver fibrosis, that ALKBH5 instigated Patched 1 (PTCH1) activation, a negative regulator of the hedgehog signaling pathway, suppressing HSC activation. Similarly, Chen *et al.* [49] reported that radiation could induce ALKBH5 expression, leading to *TIRAP* demethylation and subsequently activating downstream signaling pathways and HSCs.

Given the central role of mitochondrial metabolism in the onset and progression of chronic liver diseases [99], researchers have elucidated the involvement of m6A modification in the governance of mitochondrial function and its repercussions on hepatic fibrosis. Peroxiredoxins (PRDXs), a peroxidase superfamily crucial for inhibiting oxidative stress [100], have been investigated. Sun *et al.* [46] demonstrated that PRDX3 plays a role in restraining HSCs activation by regulating mitochondrial ROS production, thereby attenuating hepatic fibrosis. Notably, the expression of *PRDX3* was found to be controlled by YTHDF3 in an m6A-dependent manner. Furthermore, Wang *et al.* [50] reported that the loss of ALKBH5 enhances the m6A modification of Dynamin-related protein 1 (*Drp1*), leading to increased expression. *Drp1*, in turn, mediates mitochondrial fission, thereby promoting HSC proliferation and migration in hepatic fibrosis.

In recent years, emerging forms of programmed cell death have undergone extensive study, revealing that ferroptosis and pyroptosis are implicated in hepatic fibrosis and can be modulated by m6A modification [42, 47]. Shen *et al.* [47] demonstrated that increased m6A modification of the autophagy-related gene, *BECN1*, plays a crucial role in the mechanism of dihydroartemisinin for treating hepatic fibrosis. This mechanism involves preventing the activation of HSCs through the ferroptosis pathway. They further established that the reduction of FTO mediates dihydroartemisin-induced up-regulation of m6A levels, and YTHDF1 is the key reader responsible for maintaining the stability of *BECN1* mRNA. Shu *et al.* [42] conducted a study revealing that the METTL3/MALAT1/PTBP1/USP8/TAK1 axis promotes pyroptosis and inflammation in macrophages, leading to the activation of HSCs and their involvement in liver fibrosis.

Another mechanism involving m6A regulation in liver fibrosis was reported by Feng *et al.* [43], who investigated the impact of chronic corticosterone exposure on inflammation and fibrosis in the chicken liver. Their results suggested that m6A modification on heat shock proteins (HSPs) transcripts reduces HSPs, consequently suppressing the cytoprotective effect of HSPs in this model. Additionally, as mentioned in the pulmonary fibrosis section, the maturation of miRNA regulated by METTL3-mediated m6A modification was also found to be involved in hepatic fibrosis [44].

### Renal fibrosis

m6A RNA methylation was found to be significantly elevated in renal fibrosis, accompanied by increased expression of key m6A regulators, including METTL3, METTL14, ALKBH5, YTHDF1, and YTHDF3 [51]. It is noteworthy that the authors demonstrated that genetic knockdown and pharmacological inhibition of METTL3 by STM2457, a selective inhibitor of METTL3 catalytic activity, suppressed the TGF-β-induced fibrosis. Similar results were observed in a mouse model of UUO-induced kidney fibrosis. Furthermore, the researchers observed that TGF-β enhances m6A methylation of the *NET1* transcript in the vicinity of its transcription termination site, thereby promoting mRNA stability and contributing to the progression of renal fibrosis. These findings highlight the pivotal role of m6A modification in renal fibrosis.

Additionally, Ni *et al.* [52] reported the hyperactivation of METTL3 and m6A modifications in TGF-β-induced HK-2 cells and the UUO-induced mouse model. The researchers also identified another target of METTL3-regulated m6A modification, *Ena/VASP-like* (*EVL*) mRNA, which was stabilized by METTL3-induced m6A modification. In this study, the authors identified a TCM monomer, isoforsythiaside that can inhibit METTL3 activity using molecular docking and virtual screening. They found that pharmacological inhibition of METTL3 activity suppressed *EVL* m6A and subsequently reduced its interaction with Smad7.

The differential expression of m6A regulators has led to bioinformatic investigations to predict pivotal genes that could serve as diagnostic biomarkers for renal fibrosis and identify potential drug targets through machine-learning models [52]. For instance, Xing *et al.* [101] identified increased expression of *YTHDF1* in human fibrotic kidneys. They established a correlation with *Yes-associated protein* (*YAP*), a pivotal regulator of ECM production triggered by myofibroblast transformation. Their experiments in mice and cultured cells supported the role of YTHDF1 in promoting renal fibrosis by regulating *YAP*. The co-location and specific binding of YTHDF1 to *YAP* were further confirmed through immunofluorescence and RNA-binding protein immunoprecipitation. Nonetheless, YTHDF1 has also been reported to play a role in mitigating renal interstitial fibrosis induced by diabetic nephropathy by enhancing the stability of *nuclear receptor-binding SET domain protein 2* (*NSD2*) through METTL3-mediated m6A modification [56].

The significance of the m6A eraser in regulating renal fibrosis is noteworthy. However, the involvement of various m6A regulators and their specific downstream target genes can lead to divergent regulatory outcomes. Ning *et al.* [53] demonstrated that genistein promoted the restoration of ALKBH5 decline and reduced renal fibrosis, possibly by regulating EMT-related factors in an m6A-dependent manner. However, in an ischemia–reperfusion injury (IRI) model, Chen *et al.* [58] reported that ALKBH5 knockout alleviated IRI-induced renal fibrosis. Another m6A eraser, FTO, also contributes to renal fibrosis by regulating different mRNAs [57, 59]. Zhang *et al.* [57] showed that FTO reduced the m6A modification of *KCNK5*, leading to the up-regulation of TWIK-related acid-sensitive K(+) channel-2 (TASK-2, encoded by *KCNK5*) and promoting renal fibrosis. Meanwhile, Yang *et al.* [59] demonstrated that the decrease in FTO expression was associated with the anti-fibrotic effect of canagliflozin, achieved by stabilizing *SQSTM1* mRNA.

Furthermore, m6A modification of non-coding RNA has also been implicated in the development and progression of renal fibrosis. In animal and cell culture models of renal fibrosis, m6A demethylase FTO has been shown to reduce the m6A decoration of lncRNA *GAS5*, promoting the EMT process [60]. Liu *et al.* [61] unveiled that lncRNA *MALAT1* serves as a miRNA sponge, exerting inhibitory effects on *miR-145*, which, in turn, attenuates FAK expression, ultimately culminating in the promotion of EMT and renal fibrosis. Furthermore, they discovered that METTL3 plays a role in the upregulation of MALAT1. Similarly, METTL3 was found to fulfill a pivotal catalytic role in m6A modification in renal fibrosis, instigating obstructive renal fibrosis by promoting the maturation of *miR-21-5p*, consequently activating the SPRY1/ERK/NF-kB signaling pathway [54].

### Cardiac fibrosis

Cardiac fibroblasts are the critical cells involved in myocardial fibrosis. The relationship between m6A modification and cardiac fibrosis has been mainly focused on the regulation of cardiac fibroblast activation. Numerous studies have documented that the m6A methyltransferase METTL3 can induce cardiac fibroblast activation in various mechanisms, ultimately promoting myocardial fibrosis [55, 62, 63]. For instance, Li *et al.* [63] discovered that METTL3-mediated m6A modification promotes cardiac fibroblast activation and collagen accumulation, and silencing METTL3 attenuates cardiac fibrosis, possibly through the regulation of m6A modification of collagen-related genes. Zhou *et al.* [62] revealed that enhanced glycolysis was crucial for fibroblast proliferation, and METTL3 could boost glycolysis by repressing the androgen receptor, which interacts with the glycolysis marker HIF-1α, inhibiting its activation. Ding *et al.* [55] identified a differentially expressed gene, *IGFBP3*, in cardiac fibrosis and demonstrated that its knockdown suppresses the migration and proliferation of cardiac fibroblasts. In their study, the researchers observed abnormal m6A modification patterns in models of cardiac fibrosis. They also demonstrated that silencing *METTL3* could downregulate the expression of *IGFBP3*. Moreover, *Tenascin-C*, an ECM modulator implicated in cardiac fibrosis, was reported to be stabilized by METTL3-mediated m6A modification [64]. In their study, Zhuang *et al.* [65] investigated the upstream regulatory factor of METTL3 in cardiac fibrosis. They showed that the lncRNA *MetBil* protects METTL3 from degradation through the ubiquitin-proteasome pathway, thereby promoting cardiac fibrosis.

In line with the association between m6A levels and myocardial fibrosis, multiple studies have also reported the involvement of the m6A demethylase FTO in the attenuation of myocardial fibrosis. Li *et al.* [66] demonstrated that *circCELF* reduces the m6A modification of *DKK2* by up-regulating FTO expression, thereby suppressing the binding of *miR-636* to *DKK2* and increasing the expression of *DKK2*, which plays an anti-fibrotic role in cardiac fibroblast activation by inhibiting the Wnt/β-catenin signaling pathway [68].

Mathiyalagan *et al.* [102] discovered that decreased FTO expression increased m6A modification and was associated with cardiac fibrosis and enhanced angiogenesis after myocardial infarction. On the other hand, in heart failure with preserved ejection fraction, Liu *et al.* [69] demonstrated that exercise increased total m6A levels and decreased FTO expression. Overexpression of FTO reversed the benefits of exercise, leading to enhanced myocyte apoptosis, myocyte hypertrophy, and myocardial fibrosis.

Additionally, the m6A demethylase ALKBH5 has been linked to the development of cardiac fibrosis by influencing the macrophage-to-myofibroblast transition (MMT) [70]. In this study, increased expression of ALKBH5 was observed during angiotensin II-induced MMT, while macrophage-specific knockout of ALKBH5 suppressed this transition and subsequently inhibited cardiac fibrosis. Furthermore, *interleukin-11* (*IL-11*) was identified as a target of ALKBH5-mediated m6A demethylation through RNA immunoprecipitation sequencing, where ALKBH5 enhanced *IL-11* mRNA stability and elevated its protein levels.

Aside from the m6A writer and eraser, Peng *et al.* [71] reported that the lncRNA *Airn* inhibited cardiac fibroblast activation, alleviating diabetic cardiac fibrosis. Mechanistically, *Airn* stabilized *p53* mRNA in an m6A-dependent manner through the recognition of the m6A reader IGF2BP2, resulting in cardiac fibroblast cell cycle arrest and the suppression of cardiac fibrosis.

Collectively, the existing studies suggest that m6A modification plays a role in promoting cardiac fibrosis. This highlights the potential of key regulators of m6A modification as targets for treating cardiac fibrosis. Nevertheless, it is crucial to consider the widespread occurrence of m6A modification and its multifaceted functions, as a broad spectrum often signifies potential adverse impacts on the functionality of other tissues and organs. Further research is required to understand the various roles of m6A in the heart comprehensively.

### Others

Numerous studies have investigated the role of m6A modification in fibrosis affecting various organs and tissues. Liu *et al.* [67] conducted a bioinformatics analysis to identify differentially expressed mRNA transcripts with unique m6A modification patterns in hyperplastic scars compared to normal skin. Notably, these modified m6A genes were found to be significantly enriched in pathways related to fibrosis.

In the study of vitreoretinal fibrosis, a condition characterized by abnormal growth of fibrous tissue within the vitreous of the eye, Ma *et al.* [103] observed a decrease in levels of METTL3 and a reduction in total m6A abundance. Importantly, increasing the expression of METTL3 reduced the EMT induced by TGF-β1, thus ameliorating proliferative vitreoretinopathy. Conversely, in the context of subretinal fibrosis, which is characterized by fibrotic changes in the subretinal space, METTL3 was observed to facilitate fibrosis progression. This effect was achieved through the induction of m6A modification in *high mobility group AT-hook 2* (*HMGA2*), ultimately leading to the activation of SNAIL, a transcription factor known for inducing EMT [104].

Additionally, Li *et al.* [105] discovered a correlation between m6A modification and arecoline-induced oral submucosal fibrosis. They found that arecoline, a compound frequently linked to this condition, enhanced m6A levels by modulating the TGF-β signaling pathway. Consequently, they identified *MYC* as a downstream target of m6A modification, a process regulated by METTL14. Importantly, most of these published studies have primarily focused on assessing global changes in m6A levels and alterations in key m6A regulators, while the specific downstream targets remain an area ripe for exploration.

## Conclusion and perspective

Research has shown that fibrosis progression often leads to organ dysfunction, with the lung, liver, kidney, and heart being extensively studied examples. Recent research has unveiled the pivotal regulatory role of m6A modification in fibrosis processes across various organs. Importantly, the principal m6A regulators, encompassing writers, readers, and erasers, are all implicated in the regulation of organ fibrosis. This widespread involvement can be attributed to the ubiquitous and abundant nature of m6A modification. However, it is crucial to acknowledge that the mechanisms of fibrosis, mediated by m6A regulators in different organs, are intricate and subject to variation due to distinct tissue and cellular environments [83]. As highlighted in the “m6A in organ fibrosis” section, even within the same organ, similar types of m6A regulators may produce diverse regulatory outcomes in fibrosis by interacting with specific downstream target RNAs.

Expression patterns of m6A regulators are primarily context-dependent and influenced by a myriad of factors. The distribution and functionality of m6A regulators differ among various cell lines [106]. For instance, METTL3 is mainly localized in the nucleus, where it forms a complex with other writers [107]. However, a fraction of METTL3 can localize to the cytoplasm and interact with downstream RNA independently of its catalytic activity, effectively functioning as an m6A reader rather than a writer [108, 109]. Additionally, the activation of m6A regulators depends on the specific cellular environment. Some m6A readers are constitutively active, while others are induced by distinct stimuli, such as heat shock-induced up-regulation and translocation of YTHDF2 or infection-induced overexpression of YTHDF1–2 [110, 111]. Furthermore, the functional impact of m6A modification on organ fibrosis occurs through interactions with specific downstream RNAs, each playing unique roles. Therefore, it is not surprising to observe divergent regulatory effects on fibrosis by the same m6A regulators.

Despite the encouraging potential of m6A modulation in the context of fibrosis, several significant challenges must be addressed prior to its clinical translation. These include the potential for off-target effects and the difficulty of efficiently delivering m6A modulators to individual transcripts. To address these challenges, novel strategies are being developed. For example, CRISPR-Cas13b-based delivery systems are being investigated for their capacity to precisely target and modify m6A modifications on specific transcripts, thereby improving specificity and reducing off-target effects [112, 113]. Furthermore, developments in nanoparticle-based delivery systems are intended to augment the efficacy of m6A modulator delivery to intended cells [114]. It is imperative that these challenges be addressed through such advanced strategies if m6A modulation is to be successfully applied in a clinical context.

The investigation of m6A in organ fibrosis presents a promising avenue for translational research, offering new possibilities for therapeutic intervention. However, the realization of this potential will necessitate not only further preclinical and clinical studies but also robust interdisciplinary collaboration. Engaging experts in molecular biology, pharmacology, clinical medicine, and engineering materials will be crucial for overcoming current challenges and advancing m6A-targeted therapies from bench to bedside.

**Key Points**

- Fibrosis is a common consequence of chronic tissue injury affecting multiple organs, including the lung, liver, kidney, and heart, posing a major challenge to human health.
- The m6A modifications involve three classes of proteins: writers, erasers, and readers, which are involved in adding, removing, and recognizing m6A marks, respectively.
- N6-methyladenosine (m6A) RNA modifications play a key regulatory role in fibrosis in different organs, and the modifications can affect RNA structure, stability, degradation, and intracellular interactions.
- The m6A modification has great potential as a therapeutic target for fibrosis in chronic diseases.

## Data Availability

The data supporting this review are from previously reported studies and datasets, which have been cited. The processed data are available from the corresponding author upon request.
